# Approaches for sustainable professional skill development for vocational education students in Thailand

**DOI:** 10.12688/f1000research.146802.1

**Published:** 2024-04-26

**Authors:** Naksit Sakdapat

**Affiliations:** 1 Faculty of Humanities, University of the Thai Chamber of Commerce, Bangkok, Bangkok, Thailand

**Keywords:** Self-development, professional skills, sustainable development, skill development, self-improvement, problem solving skills, experiential learning.

## Abstract

**Background:**

This article presents a research study that aims to explore sustainable approaches for developing professional skills in vocational education students in Thailand. This is the second phase of the research, which utilizes a qualitative research methodology.

**Methodology:**

The key informants in this study are administrators of vocational education institutions, teachers, and students currently enrolled in vocational education institutions in Thailand, totaling 36 participants. The research uses a purposive sampling method and snowball sampling method. Data collection methods include document analysis, in-depth structured interviews, and observation. The results of the interviews are analyzed, and the content analysis is summarized. The research process consists of 4 steps: 1) literature review, 2) data collection, 3) data analysis, and 4) verification and confirmation.

**Results:**

The research findings highlight several key considerations, including: 1) factors influencing the development of professional skills among vocational education students, such as curriculum design, support and counseling, practical training in workplaces, interactive learning, problem-solving and analytical thinking skills development, practical learning, and supportive learning environments, 2) approaches for sustainable professional skill development involve employing the appropriate approaches for self-development. The steps include setting clear goals and plans, active learning and training, technology skill development, experiential learning, problem-solving skill development, participation in professional activities, self-reflection, and continuous self-improvement.

**Conclusion:**

These approaches aim to enhance the competencies of vocational education students, ensuring quality and efficiency as part of lifelong learning and sustainable development.

## Introduction

Education is crucial for the creation of a high-quality workforce with capabilities that support national development, particularly in vocational education. The goal is to produce a skilled workforce that meets the demands of the labor market, contributes to the economic advancement of the country, and enhances the competitiveness of the nation. However, the current development of vocational education reveals that it does not align with the needs of businesses. Moreover, there is a decline in the number of students pursuing vocational education, leading to a shortage of both quantity and quality in the workforce in production and labor markets ( Office of the Vocational Education Commission, 2010). In essence, the quality-oriented goal aims to develop individuals with problem-solving abilities and professional skills. Meanwhile, the quantity-oriented goal aims to increase the workforce at the middle level to at least 60% of the total workforce ( Office of the National Economic and Social Development Council, 2022). Additionally, basic competency and professional skills necessary for work in educational institutions that offer vocational education are not acknowledged by employers. This includes a lack of skills in practical work, expertise in foreign languages and/or computer skills, and problems related to studying not aligning with the job market’s needs or choosing majors based on popular trends without considering the workforce needs of businesses ( Euapipattanakul, 2022). From the aforementioned problem, the National Economic and Social Development Plan (2023) reported on the situation of education and national development in Thailand, providing recommendations to address the issues. Two key points were emphasized: the need to expedite the development of the quality of Thai education, focusing on developing the technological and innovative skills of vocational education graduates. Furthermore, there should be attention to parental values regarding vocational education, where there is a preference for general education over vocational education ( Jedynak et al., 2021). This contradicts the direction of national development, impacting resource allocation for education significantly ( Rojananan, 2016). The report on the production status and trends in vocational education in Thailand found that the proportion of students at the professional certificate level compared to the general stream is 60:40. Notably, there has been an increase in the use of professional-level labor from 9.5% in 2007 to 11.9% in 2017, respectively. However, the Thai industrial system still heavily employs less skilled professional labor. This reveals an increase in the proportion of professional-level labor, but it does not fully align with the government’s expectations for vocational education to play a key role in developing the country, especially with the trend of promoting Industry 4.0 under the Eastern Economic Corridor (EEC) framework ( Office of the Education Council, Ministry of Education, 2021). It is necessary to use vocational education labor up to 173,705 people, but it still falls short of producing enough skilled labor (excess demand) by 55,462 people or 32%. This demonstrates that Thailand still faces challenges in producing labor that does not meet the requirements in deficient fields, as the number of unemployed exceeds the shortage in every education level. The professional certificate level has a job vacancy rate 2.1 times higher than the shortage.

Therefore, it is essential to develop students at the professional level to align with and sufficiently meet the workforce needs in both quantity and quality ( Mesintree, 2016) . This is to respond to the country’s development policies and align with the sustainable development goals set by the global community ( World Bank, 2021). Increasing access to education for the population is a key goal for the development of every country ( Office of the Education Council, 2021). This aligns with Sandra M. Kaiser (2000), who emphasized that the success of vocational learning is influenced by various factors, including teachers, leadership, organizational systems, organizational structures, organizational culture, organizational atmosphere, organizational unity, organizational management, mission, strategy, government support, etc. ( Chanchaona & Santichaianan, 2012).

This agrees with the National Economic and Social Development Plan No. 13 (2023-2027). Office of the National Economic and Social Development Council (2022) outlines the development of human resources towards Thailand 4.0. The emphasis is on developing analytical thinking skills and professional skills to prepare for entry into the labor market. Planning and developing human resources that support industry, technology, and innovation are crucial. This is vital for developing human resources with necessary skills for future life and work in the 21 ^st^ century, including using professional skills in the 21 ^st^ century. Developing human resources is the most critical factor for the success of every country in transforming its economic structure. Supporting factors for professional skills development include education standards ( Muldoo, 2009), skill assessment ( Treleaven & Voola, 2008), curriculum and teaching methods ( Ogbuanya & Chukwuedo, 2017).

Under the supervision of the Office of the Vocational Education Commission in Thailand, vocational education institutions consist of 433 colleges with more than ten thousand students. These institutions are a significant force in Thailand’s development. The aforementioned principles and reasons have led the researchers to be interested in the factors that affect the professional skills of students in vocational education institutions.

This study aims to explore the factors influencing the professional skills of students in vocational education institutions in Thailand. The research findings can serve as a guideline for developing sustainable professional skills of students in vocational education institutions in Thailand. This will contribute to creating a conducive learning environment for sustainable professional development, leading to suitable employment opportunities. Additionally, it can serve as a model for other countries and contribute to establishing a global network for the professional development of vocational education students in the future.

## Literature review

### Factors affecting professional learning

Kaiser, Sandra M. (2000) studied factors that influence becoming a learner of professional skills, which are related to 8 factors: Leadership, Organizational Culture, Mission and Strategy, Management Practice, Organizational Structure, Organizational System, Working Climate, and Motivation.

The concept of Self-Efficacy Theory suggests that individuals seek things that align with their needs and choose challenging activities that come from internal motivation ( Eccies, 2002). Internal motivation persists when individuals feel they have the ability and autonomy to determine for themselves, aligning with the perception of one’s abilities ( Bandura, 1986). It is an individual’s assessment of their ability to generate and manage the actions required to achieve predetermined results. The perception of one’s abilities is the most influential variable leading to behavioral change and actions, significantly influenced by the belief in their ability to predict those behaviors ( Bandura, Adams, Hardy & Howells, 1980).

### Management of learning for vocational development in different countries

In the Federal Republic of Germany, vocational education management emphasizes collaboration between educational institutions and businesses in a bilateral system. This collaboration includes jointly developing curricula, implementing learning processes that focus on knowledge, skills, and practical training. The goal is to equip learners with competencies that align with the labor market’s needs and provide career pathways within the business sector. Additionally, there is a focus on assessment and evaluation processes that support the success factors of vocational education management in Germany. These factors encompass (1) creating awareness of the value of entering the vocational education system, (2) emphasizing the importance of investing in education for future workforce development, and (3) prioritizing work-based learning (WBL) ( Solga, Protsch, Ebner & Brzinsky-Fay, 2014).

In Australia, due to the diverse backgrounds of the country, including differences in ethnicity, religion, and culture, the country is characterized as a multicultural nation. The educational management objective is to provide alternative opportunities for learners with diverse readiness and preparedness within and outside the formal education system. The Technical and Further Education Institutions (TAFE) is established by the state to manage vocational education in the country. The 7 success factors for managing vocational education in schools include (1) readiness and diversity of curricula, (2) close collaboration between educational institutions, businesses, government, and the private sector, (3) readiness and quality of educational institutions, (4) motivating institutions to provide training in preparing learners’ skills, (5) maintaining high-quality training standards, (6) ensuring learners’ quality in terms of knowledge, abilities, skills, and (7) clear directions and objectives for professional learning and career pathways for learners ( Agbola & Lambert, 2010).

In Taiwan, the country employs a comprehensive high school system that focuses on providing students with the option to explore and discover whether they want to pursue general or vocational education. The three objectives of this system are (1) to create alternatives for students who are not ready to enter the workforce or pursue traditional education systems, (2) to integrate academic and vocational education at the high school level to create a better learning environment and more educational opportunities, and (3) to reduce the differences in the learning systems for students ( Huang & Lee, 2012).

Thailand follows the policy of vocational education development under the National Education Development Plan for Vocational Education 2017-2036. This policy, established by the Ministry of Education, aims to align learner production with the country’s development needs following the Thailand 4.0 policy. The focus is on developing learners’ professional competencies, including (1) the ability to apply knowledge, (2) the ability to apply skills in their respective professions, (3) the ability to adapt to professions, and (4) the ability to work collaboratively and develop professional skills ( Moenjak & Worswick, 2003).

Upon reviewing the literature used in this research, it is evident that education policies supporting vocational education in nearly every country are typically within the Ministry of Education. This may be a unit within the ministry, serving as a policy development framework. When combined with self-development guidelines and factors that promote professional learning, this can create a comprehensive approach to developing vocational skills, covering various dimensions.

## Methods

This research is the second phase employing a qualitative research method ( Hennink, Hutter & Bailey, 2020) to study sustainable professional skill development approaches for vocational students in Thailand. The research has received ethical clearance from The University of Thai Chamber of Commerce under the code Expedited UTCCEC/014/2565. The study involves key informants, including educational institution administrators, teachers, and vocational students totaling 36 individuals. A written consent for participation in the study has been obtained from all key informants, including educational institution administrators, teachers, and vocational students, prior to the collection of data. The key informants were selected using purposive sampling based on predefined criteria ( Ames, Glenton & Lewin, 2019) and snowball sampling ( Naderifar, Goli & Ghaljaie, 2017). The criteria include (1) administrators or directors of public vocational institutions with a minimum of 3 years of experience, (2) teachers with at least 3 years of teaching experience in vocational institutions, and (3) vocational students. Research tools include structured open-ended interviews and observations.

**Figure 1.**
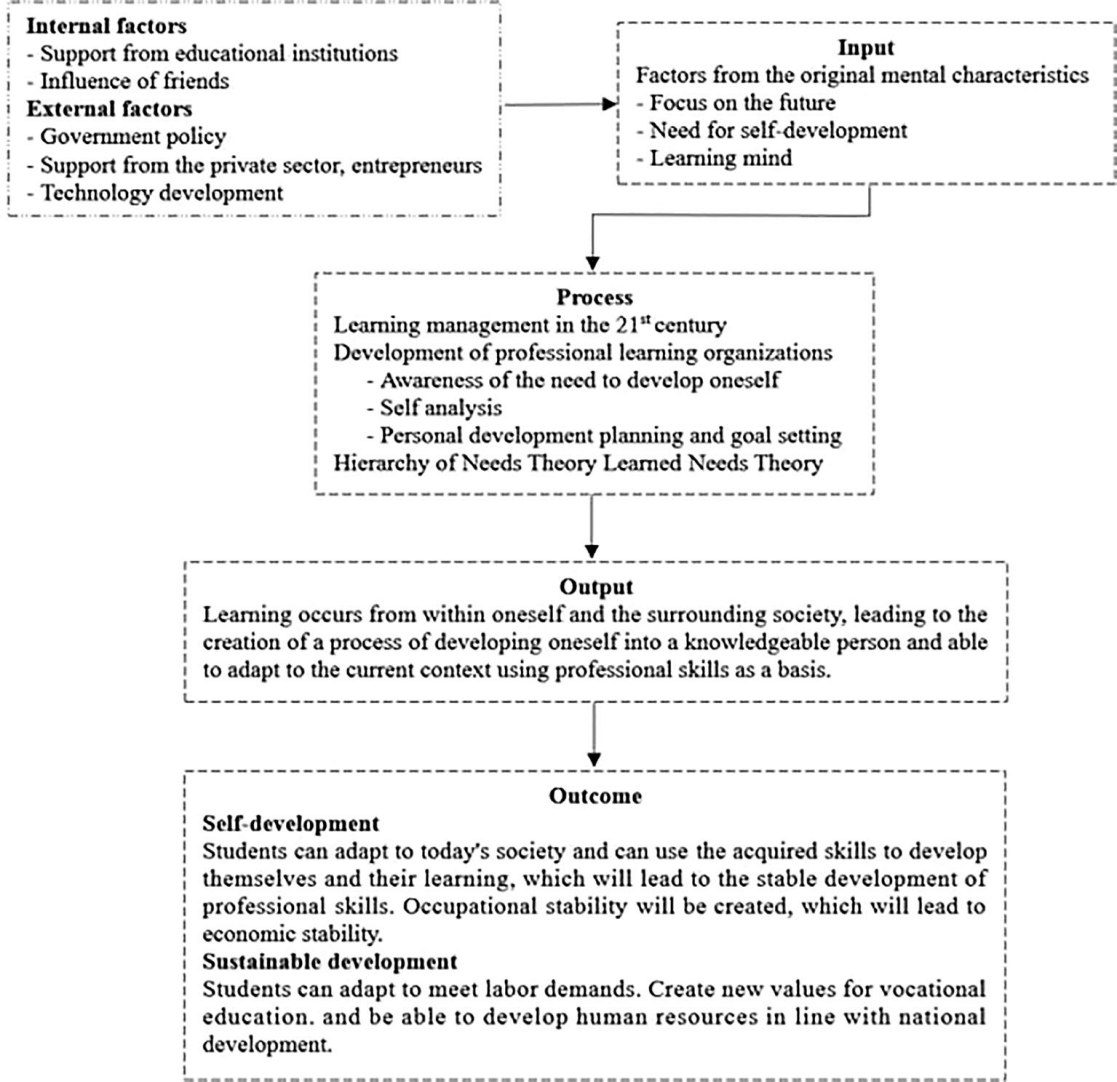
Framework and flowchart for education system focused on professional development and societal contribution.

The research process comprises 4 stages ( Figure 1): (1) Literature review and document study – The first stage involved conducting a thorough review of the existing literature and relevant documents related to sustainable professionEthical skill development approaches for vocational students in Thailand. This literature review helped to identify the key concepts and theories that are relevant to the research question. (2) Data collection and identification of relevant components – The second stage involved data collection through structured open-ended interviews and observations. The key informants, including educational institution administrators, teachers, and vocational students totaling 36 individuals, were selected using purposive sampling based on predefined criteria. The data collected from the interviews and observations were then analyzed to identify the relevant components related to sustainable professional skill development approaches. (3) Analysis and synthesis of administrators’ roles – The third stage involved the analysis and synthesis of administrators’ roles in sustainable professional skill development approaches. The data collected were analyzed using content analysis, categorizing data into four groups and indexing. The meaning of the discovered data was explained and analyzed in terms of structural features. This helped to identify the key roles of administrators in sustainable professional skill development approaches. (4) Data analysis using content analysis - The final stage involved data analysis using content analysis, categorizing data into four groups and indexing. The interpretation of the meaning of the data led to new data compilation to connect concepts and theories used as a framework according to the objective. After that, the verification and confirmation were performed. This helped to verify the findings of the study and confirm the effectiveness of the sustainable professional skill development approaches identified in the study ( Sakdapat, 2024).

### Research findings

The development of vocational students’ professional skills in vocational institutions is influenced by several significant factors, affecting preparedness for various vocational professions. These factors include:

1. Curriculum Design: Institutions should focus on creating learning experiences that equip students with skills and practical knowledge relevant to real-world work. However, interviews with students indicate that, following the COVID-19 situation, curricula have not been adjusted to align with the practical aspects of learning, leading to a gap in skills. Students express the need for more diverse courses, such as entrepreneurship or new technologies, to better meet their learning needs.
*“I want the college to offer more than this or ask students about their interests, such as becoming an entrepreneur, new technologies, because many of my friends have to study online or find books to read, incurring additional expenses.”* – Interviewee A
  Currently, students’ needs are diverse and universities should develop curricula that align with these needs. Even though vocational education curricula are adjusted every five years, the content of the subjects remains largely unchanged or undergoes minimal modifications, making the education system outdated and unresponsive to the country’s needs. Vocational education programs should not overly emphasize general foundational competencies. Instead, they should focus on practical subjects to facilitate experiential learning, aligning with the requirements of the industrial sector.
2. Support and Counseling: Students believe that appropriate guidance and support from experienced teachers and senior peers help them develop skills and knowledge. Interviews with students reveal that advice from alumni who have completed their studies, guidance on work, and self-development contribute to a better understanding of how to develop oneself.
*“Initially, I did not know what to do after graduation and joined the program following my friends. However, when I heard advice from senior students about working and self-development, it helped me understand what I should do. Now, I am learning English to get a higher salary.”* – Interviewee B
  This shows that advice from experienced individuals in the field plays a crucial role in helping students plan for their future.
3. Internship in Business Organizations: Students believe that having the opportunity to intern in various organizations helps them gain experience and understanding of the specific professional field they are interested in. While there are mandatory courses related to internships, students express a desire for additional elective courses tailored to their interests. This aligns with the institution’s management to coordinate with the private sector to increase opportunities for internships or real-world work.
*“I would like the university to have internship programs abroad because Thai vocational students are capable, and it would also help expand job opportunities. For example, in countries like Korea and Japan where salaries are high. I personally would like to work legally in those countries, so I hope the university can initiate such programs.”* - Interviewee C
4. Problem-Solving and Analytical Thinking Skills Development: Input from teachers and administrators reveals that systematic thinking is considered a crucial factor in education. However, this process takes time to yield results, and sometimes, students may become discouraged, requiring the institution to find additional courses or activities to foster students’ development.
*“The traditional vocational education format focused on skill mastery, leading to the retention of traditional approaches in curriculum or teaching methods. However, the university has now collaborated with private factories, organized competitions, allowing students to practice critical thinking in real-world work environments.”* - Interviewee D
  Developing systematic thinking and presenting reasoned ideas enables efficient problem-solving in real-world situations.
5. Practical Learning: Opportunities for hands-on experimentation, trial and error, are emphasized contributing to learning from experience and adapting to failures in practice ( Sakdapat, 2023). The interviewees provide additional insights, stating that through internships or practical courses. They discovered persistent errors that needed correction. In the classroom, this experience is viewed positively as there are teachers and peers to provide assistance. However, once in the workforce, such support may not be readily available.
6. Supportive Learning Environment: The learning environment and facilities that foster learning, such as convenient learning spaces, technology used to support learning, and opportunities to access necessary information, significantly impact learning. According to the information provided, the classroom atmosphere, including learning equipment, greatly influences the learning experience. Having sufficient computers, up-to-date and modern facilities in universities, and updating technology in industrial workshops or introducing new tools contribute to awakening students’ awareness and increasing interest. Administrators and teachers share the opinion that there is an intention to develop modern tools, but there is a budget constraint. Therefore, students must collaborate to maintain and care for various equipment for future generations to use.

As for sustainable professional skill development for vocational education students in Thailand, surveys and document analysis reveal the needs, problems, and obstacles affecting the development of students’ professional skills. The following are identified approaches for sustainable development of students’ professional skills:

Developing flexible and adaptive skills: Schools should promote collaboration with external organizations to provide diverse work-related skills. Integration of knowledge from various disciplines, such as technology and essential languages, is essential. Preparing for unforeseen changes and simulating various situations allows learners to apply knowledge to real situations, promoting readiness for unexpected changes. This is a crucial foundation for self-development towards sustainability.

Self-initiation and self-care skills: Educational institutions must provide facilities and allocate spaces to promote learning and creative thinking. Creating an environment and learning tools is vital for stimulating student learning. Punctuality, innovation, community learning, thinking about others, and contributing to society should be promoted. In the context of work, vocational students may become entrepreneurs, employees, or government officials, all of whom have roles and importance in society. Considering others and applying ethical principles is another approach that should be promoted to students for long-term responsibility toward society.

Social and Intercultural Skills under Global Societal Changes: Educational institutions need to collaborate with organizations from different countries to develop curricula that promote communication and social skills across cultures. Teachers should provide opportunities for students to develop communication and social skills, such as student exchange programs. Students should understand the role of cultural differences as a fundamental aspect of coexistence. Emphasizing the global citizenship role, which highlights the importance of everyone contributing to the development of society, making decisions, and responding to challenges at both local and global levels, is crucial.

Work Performance and Responsibility Skills are assessable. Schools and teachers must be role models for work performance and responsibility in practice. Students should actively participate in planning, execution, and problem-solving, fostering a sense of responsibility in their work. Students should be trained to think in management perspectives, considering ethical principles while working, and participating in business practices that prioritize long-term sustainability. This includes active involvement in societal development and environmental conservation, aligning with the principles of social responsibility.

Leadership and Responsibility Skills: Educational institutions should collaborate with other educational organizations, businesses, and alumni to promote students’ leadership qualities and create a network for collaborative work. Students must be self-aware and proactive in planning and problem-solving. They should be open to listening to others’ opinions and train themselves to think critically and access various perspectives when addressing different issues ( Sakdapat, 2022). Learning from real-world data and utilizing one’ professional learning abilities to analyze systematically will enable individuals to perceive the multidimensional aspects of problems. This approach goes beyond viewing problems from a single dimension, allowing students to develop themselves into leaders who are self-reliant and supportive of others. This capability aligns with the desired outcome of fostering sustainable personal development in the 21 ^st^ century.

## Discussion and Conclusions

The sustainable development of vocational skills for vocational students is a developmental approach that requires collaboration from all sectors. This includes students themselves, who must begin developing themselves to align with the lifelong learning model. The ability to rely on oneself and build sufficient resilience to cope with changes in various aspects, such as social, economic, or experiential learning in workplaces, is crucial. Learning and self-development through collaboration with others for knowledge exchange, cultivating a learning attitude through the development of tools or learning methods beneficial to oneself, and reflecting on learning outcomes and self-assessment all contribute to recognizing progress, areas for improvement, and setting goals for short and long-term professional skill development.

In terms of curriculum design, the educational structure, and policies, there should be a continuous review of the curriculum, making adjustments to suit the changing context and addressing the needs of the labor market and global changes. Emphasis should be placed on environmental awareness, ethical practices, transparency in work, the use of technology and innovation to facilitate easier access to knowledge, and the creation of professional networks spanning across public, private, and governmental sectors. Furthermore, the sustainable development of vocational skills requires determination and unwavering commitment. Consistent effort and adequate time for practice and skill improvement are essential factors in developing vocational skills at the vocational education level. The impact of skill development extends beyond individual sections of society; it elevates the education system, sets standards for society, and fosters positive attitudes toward vocational education. This leads to the development of human resources on a broader scale. Vocational education institutions play a crucial role in vocational training. Administrators and teachers are pivotal in the direction of reforming academic work, quality development, behavioral changes, and providing opportunities for collaborative thinking and work with students. Creating a learning society, offering suitable and effective learning resources, and facilitating a conducive environment for collaborative learning contribute to the overall development of vocational skills.

In the context of Thailand, the Office of the Vocational Education Commission has set guidelines for the development of quality in vocational education for students, emphasizing the importance of driving curriculum development, including measurement and evaluation, towards enhancing the quality of learners ( Office of Vocational Commission, 2018). The government recognizes the significance of driving the economy by emphasizing vocational education. During the years 2017-2023, various events led to uncertainties both in Thailand and globally. This included economic uncertainties on a global scale and the impact of pandemic situations that affected the lifestyles of people worldwide. It is an era where the world has to face unpredictable events, known as the VUCA or VUCA World, the world characterized by Disruptive Technology. The dimension of preparing the population to cope with change is greatly challenged ( LeBlanc, 2018). When considering the patterns of vocational education management in Thai and Taiwanese schools, similarities in policies can be observed. Both countries follow a comprehensive high school model, emphasizing that students explore and discover whether they want to pursue general or vocational education. In Taiwan, there is a blending of both curriculum types to stimulate overall educational development. The 3 main goals are (1) to provide options for students not ready for vocational or traditional academic paths, (2) to integrate academic and vocational education to create a better learning environment and increase educational opportunities, and (3) to reduce disparities in the learning systems of students. Thailand can adopt these approaches to adapt and develop teaching and learning methods that align with the needs of learners.

To align with the sustainable development context, the development model that meets people’s needs should have a strategic national relationship. This involves creating competitiveness in development, enhancing and cultivating human resources, and promoting social opportunities and equality. The development of the country’s economy necessitates a focus on the development of science, technology, and research to accelerate the creation of knowledge in science, technology, and innovation. This should go hand in hand with developing individuals through tertiary education institutions, which need to collaborate with research and development institutions to become Future Changers, the driving force in preparing Thais for the 21 ^st^ century. It is crucial to drive economic structural reforms that focus on values and play a crucial role in transitioning towards an innovative-based economy. Therefore, it is necessary to adapt and change roles and missions simultaneously with enhancing the potential of both higher education and vocational education institutions. Developing personnel or students at the vocational education level is about developing the country’s human capital to have the capacity and readiness to lead life in the 21 ^st^ century, contributing significantly to the country’s development, stability, and sustainability. This aligns with the government’s goal of developing human capacity to respond to the country’s development and create competitiveness on the global stage, stimulating grassroots economic growth towards Thailand 4.0. It builds confidence and creates the capability to compete globally, both in the short and long term. The government, therefore, has policies to integrate science, research, and innovation to drive the country’s development in the economic, community, and social sectors. It emphasizes the commercial application of science, research, and innovation in agriculture, industry, and services for maximum efficiency. It also emphasizes the importance of social innovation and innovation in the local context to solve problems and create opportunities. This is concurrent with developing human capital in both academic and high-level vocational aspects, preparing them for the digital age and driving Industry 4.0. This aligns with sustainable development goals 4, 8, and 9 in the 2030 Sustainable Development Agenda ( FUND, 2015).

### Recommendations

The results of the sustainable development of vocational skills among vocational education students in Thailand can lead to the following recommendations based on the research:

1. Education Standards Impact on Learning Skills and Innovation: Education standards have an impact on students’ learning skills and innovation in vocational education. Schools should have policies to contribute to the development of creativity and innovation in students. Additionally, there should be standards for evaluating educational outcomes, using assessments based on students’ work and diverse evaluation methods.
2. Curriculum and Teaching Methods Impact on Learning Skills and Innovation: The curriculum and teaching methods have an impact on students’ learning skills and innovation. Educational institutions should design curricula that bridge the gap between students and the needs of employers. This should involve adopting new teaching formats that align with the present and future eras, considering the integration of students’ knowledge to solve real-world problems through teaching methods. This will facilitate experiential learning for students.

### Ethics and consent

The research has received ethical clearance from The University of Thai Chamber of Commerce under the code Expedited UTCCEC/014/2565, and the research was approved on November 27, 2023. The study involves key informants, including educational institution administrators, teachers, and vocational students, totaling 36 individuals. Written consent for participation in the study has been obtained from all key informants, including educational institution administrators, teachers, and vocational students, prior to the collection of data. They were informed of the scope of the research, and they were also given the choice to withdraw from the study at any time. All the participants completed the study, and the researchers’ assured participants that the details will be kept confidential. The study adheres to the principles of the Declaration of Helsinki.

## Data Availability

Figshare: Approaches for Sustainable Professional Skill Development for Vocational Education Students in Thailand. https://doi.org/10.6084/m9.figshare.24924297.v2 The project contains the following underlying data: The data consists of testimonials from the participants, sharing their experiences and perspectives on the benefits of attending the vocational school. Topics covered include fields of study, reasons for choosing the school, scholarships, and job opportunities post-graduation. Data are available under the terms of the Creative Commons Attribution 4.0 International license (CC-BY 4.0). ***Reporting guidelines*** Figshare: SRQR Checklist for Approaches for sustainable professional skill development for vocational education students in Thailand, https://doi.org/10.6084/m9.figshare.24924297.v2 Data are available under the terms of the Creative Commons Attribution 4.0 International license (CC-BY 4.0).
