# Supplementary material for: Approaches for sustainable professional skill development for vocational education students in Thailand
Source: F1000Res. 2024 Apr 26;13:401. [Version 1] doi: 10.12688/f1000research.146802.1 (PMC11224711; doi:10.12688/f1000research.146802.1)
Supplement: Peer review history

# Reviewer report: Reviewer response for version 1

**DOI:** 10.5256/f1000research.160922.r290628

**Published:** 2024-07-04

**Authors:** Suranto Suranto

**Affiliations:** 1 Muhammadiyah University of Surakarta, Jawa Tengah, Indonesia

The results of the correction have been submitted in the article, can be found here, please pay attention and revise them according to the journal article guidelines "Minor Revision"

 1.Revision Major: for background, (write a citation, provide real condition data from national data, also provide a portrait of the problems that occur 2-3 problems, explain the reasons for the urgency of the research, and research output targets and describe plans for implementing research results).

 2. "This includes a lack of skills in practical work, expertise in foreign languages and/or computer skills, and problems related to studying not aligning with the job market's needs or choosing majors based on popular trends without considering the workforce needs of businesses (Euapipattanakul, 2022)." 

 Show data and sources, in the form of problem numbers (country or regional scope). Explain in detail the problem: 1, 2, 3 etc

 3."This contradicts the direction of national development, impacting resource allocation for education significantly (Rojananan, 2016).... "

 And Explain and describe what it means, contrary to national development in Thailand

 4. "This demonstrates that Thailand still faces challenges in producing labor that does not meet the requirements in deficient fields, as the number of unemployed exceeds the shortage in every education level. The professional certificate level has a job vacancy rate 2.1 times higher than the shortage."

 The research sample is focused on vocational school or diploma graduates?

 5. "This aligns with Sandra M. Kaiser (2000), who emphasized that the success of vocational learning is influenced by various factors, including teachers, leadership, organizational systems, organizational structures, organizational culture, organizational atmosphere, organizational unity, organizational management, mission, strategy, government support, etc. (Chanchaona & Santichaianan, 2012). " 

 Describe the solution to meet quality and quantity guarantees, at what level of education?

 6."Supporting factors for professional skills development include education standards (Muldoo, 2009), skill assessment (Treleaven & Voola, 2008), curriculum and teaching methods (Ogbuanya & Chukwuedo, 2017). " 

 Describe the solution for scope level education

 7."The aforementioned principles and reasons have led the researchers to be interested in the factors that affect the professional skills of students in vocational education institutions. "

 Emphasized, is the scope of the research at the student level or at vocational school students? and what professional skills are, in detail

 8. "Additionally, it can serve as a model for other countries and contribute to establishing a global network for the professional development of vocational education students in the future."

 Describe the problem portrait, research urgency, research scope, final target

 9.Revision for literature review: "When combined with self-development guidelines and factors that promote professional learning, this can create a comprehensive approach to developing vocational skills, covering various dimensions." 

 Good idea, but provide article citations that are relevant and appropriate to the study, at vocational schools or universities, as well as the expected criteria for professional skills in the 4.0 era

 10.Revision for Methods: "This research is the second phase employing a qualitative research method (Hennink, Hutter & Bailey, 2020) to study sustainable professional skill development approaches for vocational students in Thailand."

 If you are discussing vocational school students, you don't need to discuss university students. Please detail the sample or population in your research and what research approach you use?

 11."Research tools include structured open-ended interviews and observations." 

 Using 36 as a sample, is that enough? then the sampling method and explain the criteria for skilled and professional

 12."For Supportive Learning Environment: " 

 Have the 6 stages been tested as model steps or not? explain

 13."This approach goes beyond viewing problems from a single dimension, allowing students to develop themselves into leaders who are self-reliant and supportive of others. This capability aligns with the desired outcome of fostering sustainable personal development in the 21st century." 

 Explain the skilled, professional criteria desired for graduates by companies or users, of course based on user preferences or other data

 15.For References: (a) cite more articles from Scopus journals, (2) for each article cited, a DOI address is given, (3) writing articles follows the guidelines of journals

Is the work clearly and accurately presented and does it cite the current literature?

Yes

If applicable, is the statistical analysis and its interpretation appropriate?

Partly

Are all the source data underlying the results available to ensure full reproducibility?

Partly

Is the study design appropriate and is the work technically sound?

Partly

Are the conclusions drawn adequately supported by the results?

Partly

Are sufficient details of methods and analysis provided to allow replication by others?

Yes

Reviewer Expertise:

My expertise is in the field of (1) vocational education, (2) entrepreneurship

I confirm that I have read this submission and believe that I have an appropriate level of expertise to confirm that it is of an acceptable scientific standard, however I have significant reservations, as outlined above.

# Reviewer report: Reviewer response for version 1

**DOI:** 10.5256/f1000research.160922.r297903

**Published:** 2024-07-03

**Authors:** Kittisak Jermsittiparsert

**Affiliations:** 1 Faculty of Education, University of City Island, Famagusta, Cyprus; 2 Shinawatra University (Ringgold ID: 65167), Mueang Pathum Thani District, Pathum Thani, Thailand

The topic of this research is interesting and useful, both academically and practically. However, there are limitations that make this article's proposal a question of whether and to what extent it is the correct answer.

 The biggest question is selecting the 36 key informants. Will the answers received from them be answers that actually lead to sustainable professional skill development? What are the clear and appropriate criteria for selecting answers that are of sufficient value, not just the opinions of administrators, teachers, or students, that are not useful? The author should pay great attention to the quality and suitability of key informants, because if they are not the ones who can provide answers to the research questions, the data obtained and analyzed will lead to inaccurate conclusions.

 The literature review also requires a synthesis of relevant research/case studies from other countries to outline the issues to be considered/framework of the study.

 Research results should be expanded to be more detailed and concrete, at least until they are sufficient to be considered approaches for sustainable development. A brief summary followed by an interview with a single key informant may not be comprehensive enough.

 Conclusion and discussion should expand to a broader picture beyond the case study itself. In this case, it must move away from Thailand to a broader approach to developing this skill that is discussed with case studies from other countries. Likewise, the suggestion must not be a suggestion for what Thailand needs to do, but a broad academic suggestion.

Is the work clearly and accurately presented and does it cite the current literature?

Yes

If applicable, is the statistical analysis and its interpretation appropriate?

Not applicable

Are all the source data underlying the results available to ensure full reproducibility?

Yes

Is the study design appropriate and is the work technically sound?

Partly

Are the conclusions drawn adequately supported by the results?

Partly

Are sufficient details of methods and analysis provided to allow replication by others?

Yes

Reviewer Expertise:

Political Science, Public & Private Management, International Political Economy, Social Research

I confirm that I have read this submission and believe that I have an appropriate level of expertise to confirm that it is of an acceptable scientific standard, however I have significant reservations, as outlined above.

# Reviewer report: Reviewer response for version 1

**DOI:** 10.5256/f1000research.160922.r274222

**Published:** 2024-05-29

**Authors:** Joseph Mesuwini

**Affiliations:** 1 University of South Africa, Pretoria, Gauteng, South Africa

The abstract is comprehensive. However, it is silent about the theory used in the study.

 The introduction was done with the most recent sources cited.

 The literature review was much weaker in terms of the recency of sources. Most recent source was 2014 and dated one was 1980. I asked the significance of dated sources. Most recent sources could spice your study and attract audience.

 Methods were clear. Data triangulation was not shown. The theory underlying the study was not stated in the abstract and in the manuscript.

 Results were explained and supported with comments.

 Discussion of findings was done. I feel that the researcher could have shown the link between the findings and the literature.

 What does theory say, and how does it speak to your research?

 Generally, academic effort was shown in the study.

Is the work clearly and accurately presented and does it cite the current literature?

Yes

If applicable, is the statistical analysis and its interpretation appropriate?

Not applicable

Are all the source data underlying the results available to ensure full reproducibility?

Yes

Is the study design appropriate and is the work technically sound?

Yes

Are the conclusions drawn adequately supported by the results?

Yes

Are sufficient details of methods and analysis provided to allow replication by others?

Yes

Reviewer Expertise:

TVET /WIL / WBE/WBL/ Technology education/ vocational education/

I confirm that I have read this submission and believe that I have an appropriate level of expertise to confirm that it is of an acceptable scientific standard.
